# 

**Published:** 1903-07-15

**Authors:** 


### CONTENTS.

Root-Canal Fillings, - *By John I. Hart, D.D.S.* 325

A University Degree for English Dentists, *By Wm. Guy, F.R.C.S., L.D.S.* 339

Anodyne Remedies, - - *By N. S. Hoff, D.D.S.* 351

Are the Teeth Deteriorating More Rapidly at the Present Time than in the Past, and if So, Why? *By F. H. Essig, D.D.S.* 359

National Dental Association - - - - - - 363

Removal of Pulps Painlessly, - *By W. C. Davis* 365

EDITORIAL, - - - - - - - - 367

ANNOUNCEMENTS, - - - - - - - 368

BIBLIOGRAPHY, - - - - - - - - 369

INDENTS, - - - - - - - - - - 371

